# Tracking Anxiety in Chronic Intestinal Failure: The Role of Time in Healing

**DOI:** 10.3390/healthcare13192503

**Published:** 2025-10-02

**Authors:** Lidia Santarpia, Raffaella Orefice, Lucia Alfonsi, Fabrizio Pasanisi

**Affiliations:** Internal Medicine and Clinical Nutrition, Department of Clinical Medicine and Surgery, Federico II University Hospital, 80131 Naples, Italylucia.alfonsi@unina.it (L.A.); pasanisi@unina.it (F.P.)

**Keywords:** home parenteral nutrition, HAM-A, chronic intestinal failure, caregivers

## Abstract

**Introduction:** Chronic intestinal failure (CIF) and the resulting dependence on home parenteral nutrition (HPN) often occur abruptly, profoundly disrupting the daily lives of patients and their caregivers. Both groups face persistent psychological and relational challenges, yet evidence on their long-term mental-health trajectories remains scarce. This study aimed to assess the progression of anxiety symptoms over 12 months in CIF patient–caregiver pairs, and to explore whether participation in a systemic–relational psychotherapy program could influence these outcomes. **Methods:** The Hamilton Anxiety Rating Scale (HAM-A) was administered to all participating patient–caregiver pairs, who were also invited to attend free biweekly psychotherapy sessions for one year. Pairs who accepted (Group Y) were compared with those who declined (Group N). **Results:** At baseline, both patients and caregivers in both groups exhibited high anxiety levels. Group Y (n = 10) patients had significantly higher total HAM-A scores than Group N (n = 40) patients (*p =* 0.048); a similar, non-significant trend was observed among caregivers. Emotional and somatic component scores remained largely unchanged at 6 and 12 months, regardless of group allocation. **Conclusions:** CIF imposes a substantial and persistent anxiety burden on both patients and caregivers. In this cohort, a brief systemic–relational psychotherapy program, offered quarterly, did not significantly modify anxiety levels over 12 months. These findings highlight the need for more intensive, sustained, and possibly earlier psychological interventions tailored to the dyadic experience of living with CIF and HPN.

## 1. Introduction

Living with a chronic illness such as chronic intestinal failure (CIF) is a persistent source of emotional and functional disruption for both patients and their caregivers [1]. CIF often develops unexpectedly, as a consequence of ischemic bowel disease, Crohn’s disease, or surgical complications, and requires long-term dependence on home parenteral nutrition (HPN). This condition profoundly alters autonomy, daily routines, and social roles [1,2,3], imposing not only physiological but also substantial psychological burdens. Patients must adjust to a loss of independence and a highly medicalized lifestyle, while caregivers are often thrust into complex roles that combine emotional support with demanding practical tasks—frequently at the expense of their own well-being [4,5,6,7].

The patient’s role undergoes profound changes both socially (e.g., inability to work) and within the family structure [8]. Patients frequently experience fatigue, reduced work capacity, and the constant demands of HPN therapy, which include adhering to dietary and fluid restrictions, monitoring intestinal function, and, in some cases, managing a stoma [9,10]. Dependence on HPN affects autonomy, employability, mobility, and social life, and significantly disrupts sleep when therapy is administered during nighttime hours [11,12,13,14].

Most studies in this field have focused primarily on patients, with limited attention to caregivers, whose daily routines are deeply reshaped by the need to provide ongoing support [15,16,17,18]. While the biomedical impact of CIF and HPN is well documented [7], the psychological dimension—particularly the evolution of anxiety over time and the dyadic nature of patient–caregiver burden—remains underexplored.

Recent research highlights the importance of mutual emotional regulation in long-term illness caregiving relationships and underscores the need for interventions that target both members of the dyad [19,20,21,22,23,24]. From a psychoneuroimmunological and psychosomatic perspective, chronic stress and unresolved anxiety may contribute to systemic dysregulation and reduced quality of life [25]. Evidence supports the value of dyadic psychological interventions in chronic disease management. Meta-analyses indicate that systemic or couple-based therapies can improve anxiety, communication, and emotional resilience in families coping with long-term illness. Specifically, moderate to strong effects of family-based interventions in reducing psychological distress were reported, when caregiving becomes a central component of the patient’s life [18,21,22].

In the context of HPN, where therapy is administered daily and often during the night, caregivers are not only practical assistants but also emotional co-sufferers. Systemic–relational approaches enable both patient and caregiver to explore how the illness affects their relationship and self-perception, fostering shared adaptation rather than isolated coping. Although such interventions require more time than individual therapies, their focus on the relational architecture of distress makes them particularly suited for managing chronic conditions such as CIF [26].

While individual cognitive–behavioural therapies have demonstrated efficacy in reducing anxiety in chronic illness [27,28], systemic–relational psychotherapy directly addresses the emotional dynamics within the patient–caregiver relationship. This approach aims to restore functional communication, emotional reciprocity, and relational balance—especially when chronic illness reshapes personal roles and identities [5,29,30].

Despite growing recognition of these dynamics, there is a lack of longitudinal research evaluating anxiety trajectories in both patients and caregivers with CIF, and the potential impact of dyadic psychological interventions. This study addresses that gap by assessing the progression of anxiety symptoms over a 12-month period using the Hamilton Anxiety Rating Scale (HAM-A) in CIF patient–caregiver pairs who either participated in a systemic–relational psychotherapy program or declined participation. Primary outcomes include changes in overall anxiety scores over time; secondary analyses explore specific symptom domains (e.g., tension, fear, somatization) and potential differences between patients and caregivers.

This work builds on our previous publication, which reported baseline anxiety levels in the same cohort [29,30,31]. The present study extends those findings with two follow-up assessments at 6 and 12 months, providing a longitudinal view of the psychological burden in this population.

Understanding the psychological impact of CIF on both patients and caregivers—and evaluating the potential benefits of systemic–relational psychotherapy—is particularly relevant in Italy, where family members are the primary providers of caregiving. Although the national healthcare system offers universal coverage, psychological support for patients with rare chronic conditions and their caregivers is limited. Moreover, specialized HPN centres are often located far from patients’ homes, exacerbating isolation and perceived burden. These insights may guide the development of holistic care pathways, inform healthcare policies, and contribute to tailored psychosocial interventions aimed at improving quality of life and long-term adaptation in rare chronic conditions.

## 2. Methods

### 2.1. Study Design

This was a prospective, observational, non-randomized longitudinal study conducted at a single tertiary care centre in Italy. Data were collected at three time points: baseline (T0), 6 months (T1), and 12 months (T2). The study compared CIF patient–caregiver pairs who agreed to participate in a systemic–relational psychotherapy program (Group Y) with those who declined participation (Group N).

### 2.2. Participants

All consecutive adult patients (aged 18–70 years) diagnosed with chronic intestinal failure (CIF) and receiving home parenteral nutrition (HPN) for at least one month were invited to participate, along with their primary informal caregivers. Written informed consent was obtained from all participants.

### 2.3. Instruments

Anxiety levels in both patients and caregivers were assessed using the Hamilton Anxiety Rating Scale (HAM-A), a clinician-administered tool widely used in medical–psychological research to evaluate the severity of anxiety symptoms [32,33]. The Italian version of the HAM-A, previously validated and culturally adapted for clinical use, was administered in structured interviews by trained psychologists.

The HAM-A consists of 14 items covering both somatic and psychological domains, each rated on a 5-point Likert scale (0–4) according to symptom intensity perceived by the participant: 0 = absent, 1 = mild, 2 = moderate, 3 = severe, 4 = very severe. Total scores range from 0 to 56.

The 14 items are as follows:

1. Anxious mood (worries, fear of the worst, irritability);
2. Tension (startle response, fatigability, restlessness, easy tearfulness, trembling, inability to relax);
3. Fears (of the dark, traffic, crowds, being alone);
4. Insomnia (difficulty falling asleep, interrupted or unrefreshing sleep, nightmares, night terrors);
5. Cognitive functioning (poor memory, difficulty concentrating);
6. Depressed mood (loss of interest, anhedonia, feelings of depression, mood lability);
7. Somatic muscular symptoms (aches, stiffness, bruxism, twitching, myoclonus, unsteady voice, increased tone);
8. Sensory symptoms (tinnitus, blurred vision, hot/cold flushes, weakness, paraesthesia);
9. Cardiovascular symptoms (tachycardia, palpitations, chest pain, vascular throbbing, presyncope);
10. Respiratory symptoms (chest tightness, choking sensations, sighing, dyspnoea);
11. Gastrointestinal symptoms (IBS-type symptoms, abdominal pain, bloating, dysphagia, nausea, vomiting, altered bowel habits, weight loss);
12. Genitourinary symptoms (urinary frequency/urgency, menstrual disturbances, sexual dysfunction);
13. Autonomic symptoms (dry mouth, headaches, flushing, sweating, dizziness, goosebumps);
14. Observed behaviour (fidgeting, restlessness, hand tremors, strained facial expressions, sighing, pallor).

Internal consistency for the Italian HAM-A has been reported as Cronbach’s α = 0.88 in clinical samples, indicating good reliability [34]. In our sample, baseline Cronbach’s α was 0.86.

Clinical cut-offs were applied as follows:

0–17: no or mild anxiety;

18–25: moderate anxiety;

≥26: severe anxiety.

### 2.4. Intervention and Data Collection

Following the baseline interview and initial psychological consultation, participants were invited to join a free systemic–relational psychotherapy program. The intervention consisted of bi-weekly 60 min sessions over 12 months, with both patient and caregiver attending each session. The therapeutic work focused on dyadic interaction patterns, emotional expression, and role clarification; mindfulness-based techniques were selectively integrated to support emotional regulation. All sessions were conducted by the same experienced clinical psychologist to ensure consistency.

Participants who agreed to attend the program were assigned to Group Y; those who declined formed Group N. Reasons for non-participation (e.g., logistical barriers, scepticism toward psychotherapy) were recorded qualitatively but not formally analysed.

Demographic, clinical, and social variables were obtained through structured interviews and medical records, including: age, sex, education (primary school, secondary school, high school, university degree), kinship relationship (spouse, child, parent, sibling), employment status (employed, unemployed, housewife, retired), disease duration, primary diagnosis, stoma status, and residual bowel anatomy.

### 2.5. Ethical Considerations

The study was conducted in accordance with the Declaration of Helsinki and received approval from the Ethics Committee of A.O.U. Federico II—AORN Cardarelli (approval date: 24 October 2018; protocol no. 317/18).

### 2.6. Statistical Analysis

All data were entered into a dedicated database and analysed using IBM SPSS Statistics version 28. Descriptive statistics are presented as means ± standard deviation (SD) for continuous variables and as frequencies and percentages for categorical variables.

The normality of data distribution was assessed using the Shapiro–Wilk test, and homogeneity of variances was verified with Levene’s test. For comparisons between groups, Student’s *t*-test was used for continuous variables with normal distribution; the Mann–Whitney U test was applied for non-parametric data; categorical variables were compared using the Chi-square test or Fisher’s exact test, as appropriate. A *p*-value < 0.05 was considered statistically significant. Where relevant, 95% confidence intervals and effect sizes (Cohen’s d) were calculated to assess clinical relevance.

## 3. Results

### 3.1. Patient–Caregiver Comparisons

A total of 50 patients (28 females, 22 males) and their respective 50 caregivers (34 females, 16 males), all aged between 18 and 70 years, were enrolled. Only 10 patient–caregiver pairs agreed to participate in the psychotherapy program (Group Y), while the remaining 40 pairs declined (Group N). Comparisons between groups were performed at baseline, 6 months, and 12 months.

The demographic and clinical characteristics of patients and caregivers, stratified by participation in the psychotherapy program, are reported in Table 1a (patients) and Table 1b (caregivers). No significant differences were found between Groups Y and N in patients with respect to type or duration of disease or other baseline characteristics.

### 3.2. Patient–Caregiver Comparisons (Overall Sample)

Regardless of psychotherapy participation, caregivers showed: higher baseline *fear* scores compared to patients (*t* (98) = −3.502; *p* < 0.001; 95% CI: –1.473 to –0.407; *d* = −0.700); higher *anxiety* (*t* (98) = −2.182; *p* = 0.032; 95% CI: −1.031 to –0.49; *d* = −0.436); and *tension* (*t* (98) = −2.031; *p* = 0.045; 95% CI: –0.909 to –0.011; *d* = −0.406) at 6 months; higher *observed behaviour alterations during the interview* both at 6 months (*t* (98) = −2.685; *p* = 0.009; 95% CI: –1.113 to –0.167; *d* = −0.537) and at 12 months (*t* (98) = −2.252; *p* = 0.027; 95% CI: –0.980 to –0.062; *d* = −0.460).

Conversely, patients exhibited: *higher somatic muscular symptoms* at baseline (*t* (98) = 2.385; *p* = 0.019; 95% CI: 0.111 to 1.209; *d* = 0.477) and at 6 months (*t* (98) = 2.045; *p* = 0.044; 95% CI: 0.016 to 1.064; *d* = 0.409); higher *gastrointestinal symptoms* at baseline (*t* (98) = 3.209; *p* = 0.002; 95% CI: 0.313 to 1.327; *d* = 0.642), 6 months (*t* (98) = 2.172; *p* = 0.032; 95% CI: 0.048 to 1.072; *d* = 0.434), and 12 months (*t* (98) = 2.462; *p* < 0.001; 95% CI: 0.372 to 1.337; *d* = 0.718); higher *genitourinary symptoms* at baseline (*t* (98) = 2.462; *p* = 0.016; 95% CI: 0.097 to 0.903; *d* = 0.492) and 12 months (*t* (98) = 2.220; *p* = 0.029; 95% CI: 0.044 to 0.789; *d* = 0.919).

### 3.3. Between-Group Comparisons (Group Y vs. Group N)

At baseline, total HAM-A scores were significantly higher in Group Y patients compared to Group N patients (Group Y: 26.9 ± 10.7 vs. Group N: 19.5 ± 10.3; *t* (48) = 2.025, *p* = 0.048; 95% CI: 0.053 to 14.847; *d* = 0.716). No significant differences were found at 6 months or at 12 months. In caregivers, baseline HAM-A scores were also higher in Group Y than in Group N, although not significantly. Similarly, no significant differences were observed at 6 months or at 12 months.

Analysis of individual HAM-A items revealed:

Anxiety: Baseline anxiety scores were significantly higher in Group Y patients than in Group N (*t* (48) = 3.183; *p* = 0.003; 95% CI: 0.451 to 1.999; *d* = 1.125), with no significant differences in caregivers. Anxiety levels in all four groups remained unchanged at 6 and 12 months.

Tension: Baseline tension was high in caregivers from both Groups Y and N and did not change over time. In patients, tension was significantly higher in Group Y than in Group N (*t* (48) = 2.011; *p* = 0.017; 95% CI: 0.000 to 1.800; *d* = 0.711). In Group Y patients, tension significantly decreased from baseline to 6 months (*t* (9) = 2.333; *p* = 0.045; 95% CI: 0.021 to 1.379; *d* = 0.738), with no further improvement at 12 months.

Depressed mood: Baseline scores were significantly higher in Group Y patients (*t* (48) = 2.063; *p* = 0.045; 95% CI: 0.024 to 1.826; *d* = 0.729) and caregivers (*t* (48) = 2.298; *p* = 0.026; 95% CI: 0.119 to 1.781; *d =* 0.813) compared to their respective Group N counterparts.

Sensory symptoms: Baseline involvement was greater in Group Y caregivers (*t* (48) = 3.054; *p* = 0.004; 95% CI: 0.410 to 1.990; *d* = 1.080) and Group Y patients (*t* (48) = 2.220; *p* = 0.031; 95% CI: 0.087 to 1.763; *d* = 0.785) than in their respective Group N counterparts.

No significant differences were found between groups for fear, insomnia, cognitive function, cardiovascular, respiratory, gastrointestinal, genitourinary, or autonomic symptoms, nor for observed behaviour during the interview, except for the previously noted between-patient/caregiver differences.

## 4. Discussion

Chronic intestinal failure (CIF) encompasses a heterogeneous group of disorders that differ in residual intestinal length, the presence or absence of a stoma, the average daily volume of parenteral nutrition, and the frequency of its administration. The psychological state of patients with CIF is influenced by illness awareness, the anatomical and clinical changes they experience, their dependence on home parenteral nutrition (HPN), and concerns regarding long-term prognosis [5,10,17]. Likewise, the evolving needs of the patient profoundly affect the daily routines and professional lives of caregivers, who usually assume this role not by choice but as a result of emotional and family bonds.

### 4.1. Summary of Main Findings

This study confirmed high baseline anxiety levels—measured by the total HAM-A score—in both patients and caregivers, with a tendency toward higher scores in the couples who opted for psychotherapy (Group Y). This may reflect either a more distressing initial experience of the condition or a greater emotional awareness and readiness to acknowledge psychological difficulties in Group Y. Notably, only 10 of 50 patient–caregiver pairs agreed to participate in the program. The most frequently reported reasons for declining were scepticism about psychotherapy, logistical difficulties, and lack of time.

The first six HAM-A items—covering anxiety, tension, fear, and depressed mood—were markedly impaired in both patients and caregivers, confirming that CIF and HPN represent destabilizing, chronic challenges to daily life. Although not statistically significant, caregivers consistently exhibited higher anxiety levels than patients, suggesting a greater emotional burden. Over time, patients in Group Y showed a small but significant reduction in tension from baseline to 6 months, though this improvement was not sustained at 12 months.

### 4.2. Emotional vs. Somatic Symptom Profiles

Somatic HAM-A items—muscular, cardiovascular, respiratory, gastrointestinal, genitourinary, autonomic symptoms, and observed behaviour—can be interpreted as the somatization of psychological distress. In our cohort, these symptoms were generally less pronounced than emotional ones, particularly in caregivers. Exceptions included higher muscular, gastrointestinal, and genitourinary scores in patients, likely reflecting direct consequences of CIF (e.g., abdominal pain, increased bowel sounds, nocturnal diuresis due to HPN). Cardiovascular and respiratory symptoms were mostly absent or mild across groups and time points. Sensory symptoms (e.g., tinnitus, hot flashes, chills) were significantly more frequent in Group Y caregivers and patients than in their respective Group N counterparts.

Overall, the pattern suggests that caregivers were more affected by psychological distress, whereas patients reported more somatic manifestations—possibly reflecting different coping mechanisms and sources of stress.

### 4.3. Theoretical Rationale for the Psychotherapeutic Model

The choice of systemic–relational psychotherapy was informed by both theoretical and clinical considerations. CIF and HPN disrupt identity, autonomy, and lifestyle for patients, while caregivers face a demanding, often unexpected support role. This creates a dyadic burden—a shared emotional load shaped by interactional dynamics [5,10,17,19].

Unlike individual-focused approaches such as cognitive–behavioural therapy (CBT), systemic–relational psychotherapy conceptualizes symptoms as expressions of relational dysfunction. It targets communication patterns, emotional co-regulation, and role restructuring—especially relevant when illness leads to role reversals (e.g., spouse assuming a parental role, daughter becoming a caregiver to her mother) [20,21,23]. In Mediterranean cultures, these role shifts are particularly pronounced and may foster dependency dynamics that erode the original relationship. The therapeutic process works to re-establish balanced roles, validate both parties’ needs, and encourage shared adaptation rather than unilateral sacrifice [25,35,36,37,38].

This intervention is inherently long-term; meaningful change in relational architecture often requires sustained engagement.

### 4.4. Interpretation in Light of the Results

Although anxiety scores did not significantly improve overall, the subgroup of patients who chose psychotherapy began with higher anxiety and tension scores, possibly reflecting greater psychological vulnerability but also a higher readiness to seek help. The modest decrease in tension at 6 months among Group Y patients may represent an early sign of relational adjustment, even if not sustained at 12 months. These subtle shifts—difficult to capture in standard symptom scales—are consistent with systemic therapy’s focus on deeper relational restructuring rather than short-term symptom relief.

### 4.5. Clinical Implications and Future Directions

The findings underscore the need to recognize CIF as a condition affecting both patients and caregivers in an interdependent manner. Given the persistence of emotional distress, future interventions should explore increasing accessibility to psychotherapy through telehealth or home-based formats; offering more intensive or longer-term systemic–relational programs; incorporating psychoeducation and early intervention soon after CIF diagnosis; and integrating caregiver-specific support into multidisciplinary care pathways.

Further longitudinal studies with larger samples are warranted to clarify the trajectories of both emotional and somatic symptoms and to identify predictors of therapeutic engagement and benefit.

## 5. Study Limitations

This study has several limitations that should be acknowledged.

### 5.1. Sample Size and Study Design

Due to the rarity of CIF and the variability in its clinical management, this monocentric study included a relatively small number of participants. The limited sample size—particularly in the intervention group—reduced statistical power, increasing the risk of type II errors and limiting the generalizability of results to other settings. Furthermore, the non-randomized design and the self-selection of participants into the psychotherapy program introduce a potential selection bias. Results should therefore be interpreted with caution and considered exploratory in nature.

### 5.2. Measurement Tools

The HAM-A, while widely used to assess anxiety, is a non-specific instrument that can be applied to various populations and conditions. It is not tailored to the unique characteristics of CIF patients on HPN. The use of a disease-specific or condition-adapted measure could have provided greater sensitivity in detecting relevant psychological changes.

### 5.3. Unmeasured Confounding Variables

We did not systematically collect data on psychotropic medication use, psychiatric history, or concurrent coping strategies. These factors could have influenced anxiety levels and moderated the effects of the intervention. Future research should incorporate more comprehensive baseline assessments to control for such confounders.

### 5.4. Limited Existing Literature

The available literature on the quality of life of CIF patients on HPN—and even less so on their caregivers—is scarce. Existing studies often focus on oncological or paediatric populations [23,24,25,26], use heterogeneous methodologies, and target different aspects of burden (emotional, social, or economic). Despite this, our findings are consistent with available evidence and reinforce the need to address the psychosocial dimensions of CIF and HPN [27,28,29].

### 5.5. Accessibility and Participation Barriers

Only 10 out of 50 patient–caregiver pairs participated in the psychotherapy program. Reported barriers included scepticism toward psychotherapy, logistical difficulties (e.g., distance from the treatment centre), and lack of time. Such obstacles may have prevented the inclusion of individuals who could have benefited from the intervention.

### 5.6. Duration and Intensity of the Intervention

Systemic–relational psychotherapy is inherently a long-term intervention, often requiring extended engagement to achieve tangible relational changes. The one-year follow-up period of this observational study may not have been sufficient to capture the full therapeutic impact.

## 6. Conclusions

This study underscores the need for a holistic, dyad-centred approach to the management of chronic intestinal failure (CIF) and home parenteral nutrition (HPN). Emotional distress in this context is not solely an individual experience but a shared, interdependent burden that affects both patients and their caregivers. Interventions that address the needs of both members of the dyad—such as systemic–relational psychotherapy—may foster resilience, improve relational functioning, and support long-term adaptation to the demands of CIF and HPN.

Future research should prioritize randomized controlled trials of dyadic interventions specifically tailored to the CIF–HPN population, with adequate sample sizes and longer follow-up periods. Comparative studies evaluating systemic–relational versus cognitive–behavioural approaches could clarify which therapeutic strategies yield the greatest benefits for emotional well-being, relationship quality, and quality of life in this highly vulnerable group.

## Figures and Tables

**Table 1:** Demographics and baseline characteristics of patients (**a**) and caregivers (**b**) by psychotherapy participation.

**(a)**					
**PATIENTS**					
	**Group Y (n.10)**		**Group N (n.40)**		***p***
Age (years)	41.2 ± 15.5 (21–61)		50.2 ± 14.5 (18/69)		n.s.
Gender	9 F/1 M		19 F/21 M		-
Disease duration (yrs)	7.1 ± 6.2		6.4 ± 5.3		n.s.
Primary disease	3 intestinal infarction 4 Crohn’s disease 1 intestinal volvulus 1 intestinal pseudobstruction 1 bariatric surgery		14 intestinal infarction 13 Crohn’s disease 4 intestinal volvulus 3 intestinal adhesions 1 intestinal pseudobstruction 1 bariatric surgery 2 mucosal disease 2 radiation enteritis		
Residual small bowel length, cm median (range)	108 (15–300) 1 pts no resection		25–300 6 pts no resection		
Presence of stoma (yes/no)	3/10		9/40		
Colon in continuity (% Cummings) Median (range)	70 (0–100)		70 (0–100)		
Days of infusion/week	5 (3–7)		5 (3–7)		
Education level	2 secondary school/7 high school license/1 university degree		5 primary/15 secondary school/16 high school license/4 university degree		
Employment	1 housewife/4 employed/1 retired, 4 unemployed		15 housewives/15 employed/4 retired/6 unemployed		
**Total HAM-A score**	**Group Y (10)**	**Group N (40)**	**Statistic test**	***p*-value**	**Effect size**
basal	26.9 ± 10.7	19.5 ± 10.3	*t* (48) = 2.025	0.048	*d* = 0.716
6 months	24.3 ± 8.1	18.4 ± 12.4	*t* (48) = 1.436	n.s.	*d* = 0.508
12 months	24.0 ± 10.5	20.4 ± 12.2	*t* (48) = 0.863	n.s.	*d* = 0.307
**(b)**					
**CAREGIVERS**					
	**Group Y (n.10)**		**Group N (n.40)**		***p***
Age (years)	53.6 ± 14.5 (29–71)		50.0 ± 13.0 (21–70)		n.s.
Gender	6 F/4 M		28 F/12 M		-
Education level	4 secondary school/6 high school license		3 primary/14 secondary school/17 high school license/6 university degree		
Degree of kinship	5 husband/wife 4 mothers 1 sister		25 husband/wife 6 son/daughter 7 mother/father 2 brother/sister		
Employement	5 housewives 1 retired 4 without work 3 payed work		15 housewives 3 retired 2 without work 20 payed work		
**Total HAM-A score**	**Group Y (10)**	**Group N (40)**	**Statistic test**	***p*-value**	**Effect size**
basal	25.9 ± 12.1	20.5 ± 10.4	*t* (48) = 1.429	n.s.	*d* = 0.505
6 months	22.8 ± 8.1	20.6 ± 10.3	*t* (48) = 0.614	n.s.	*d* = 0.217
12 months	21.6 ± 5.0	21.6 ± 11.4	*t* (48) = 0.034	n.s.	*d* = −0.001

## Data Availability

The data presented in this study are available on request from the corresponding author. The data are not publicly available due to privacy restrictions.
